# Conjunction of standing wave and resonance in asymmetric nanowires: a mechanism for thermal rectification and remote energy accumulation

**DOI:** 10.1038/srep17525

**Published:** 2015-12-02

**Authors:** Yue-Yang Liu, Wu-Xing Zhou, Ke-Qiu Chen

**Affiliations:** 1 Department of Applied Physics, School of Physics and Electronics, Hunan University, Changsha 410082, China

## Abstract

As an important way to control and manage heat transport, thermal rectification has become an elementary issue in the field of phononics and plays a key role in the designing of thermal devices. Here we investigate systematically the standing wave and the accompanying resonance process in asymmetric nanowires to understand the standing wave itself and its great effect on thermal rectification. Results show that the standing wave is sensitive to both the structural and thermal properties of the material, and its great effect on enhancing the thermal rectification is realized not only by the energy-localization nature of the standing wave, but also by the resonance-caused large amplitude and high energy of the standing wave.

Heat, whether in the form of energy or acting as a signal, is increasingly influential in modern industries and technologies. The need to control and manage heat has continuously driven us to look for new methods and new materials for that purpose. In contrast to heat-conducting applications that require materials with high thermal conductivity, and thermoelectric applications that require materials with low thermal conductivity, applications where heat is to be processed as information^1^ require materials with asymmetric thermal conductivities, i.e., a strong heat conduction ability in one direction and a weak heat conduction ability in the opposite direction. Theoretically, such thermal rectification effect has been found in nonlinear lattices/chains^2^,^3^,^4^,^5^,^6^,^7^,^8^,^9^,^10^,^11^,^12^, hybrid structures^13^,^14^,^15^,^16^,^17^,^18^,^19^,^20^,^21^, three-terminal junctions^22^,^23^,^24^ and diversiform asymmetric single-material structures, including carbon nanotubes^25^,^26^,^27^, carbon nanocones^28^, diamond^29^, graphene nanoribbons^30^,^31^,^32^,^33^,^34^,^35^,^36^, and polyethylene Nanofibers^37^. Experiments using carbon nanotubes^38^, quantum dots^39^, reduced graphene oxide^40^ and phase change materials^41^,^42^,^43^ have also been carried out successfully.

Recently, we managed to reveal the existence of standing wave in graded InAs/GaAs core-shell nanowires when the narrow end is in higher temperature^44^, and found that the formation of the standing wave can greatly enhance the thermal rectification effect of the nanowire. However, it is obvious that the standing wave that occurs in graded nanowires is far from being understood, and characteristics including the wavelength, frequency, amplitude and velocity have still to be studied in detail. Also, questions over aspects such as where the standing wave originates from, how the standing wave hinders heat transport so significantly, and whether the mechanism takes effect in nanowires of different structural and thermal properties, have still to be answered.

In this work, we first get a closer look at the configuration and characteristics of the standing wave in a graded InAs nanowire, and then manage to figure out how the standing wave enhances the rectification effect by checking the deformation of the nanowire and monitoring the vibration density of states (VDOS) of the nanowire before and after the temperature bias is inverted. We also find the existence of resonance in the system after noticing the energy accumulation process. After that, we present the investigation on asymmetric Silicon nanowires to show the generality of our conclusion and the uniqueness of each case. Finally, we give a discussion about the origin of the standing wave and the resonance along with the important role that structural and thermal parameters play in the issue.

## Results

### Standing wave and resonance in graded InAs nanowires

**Figure 1:**
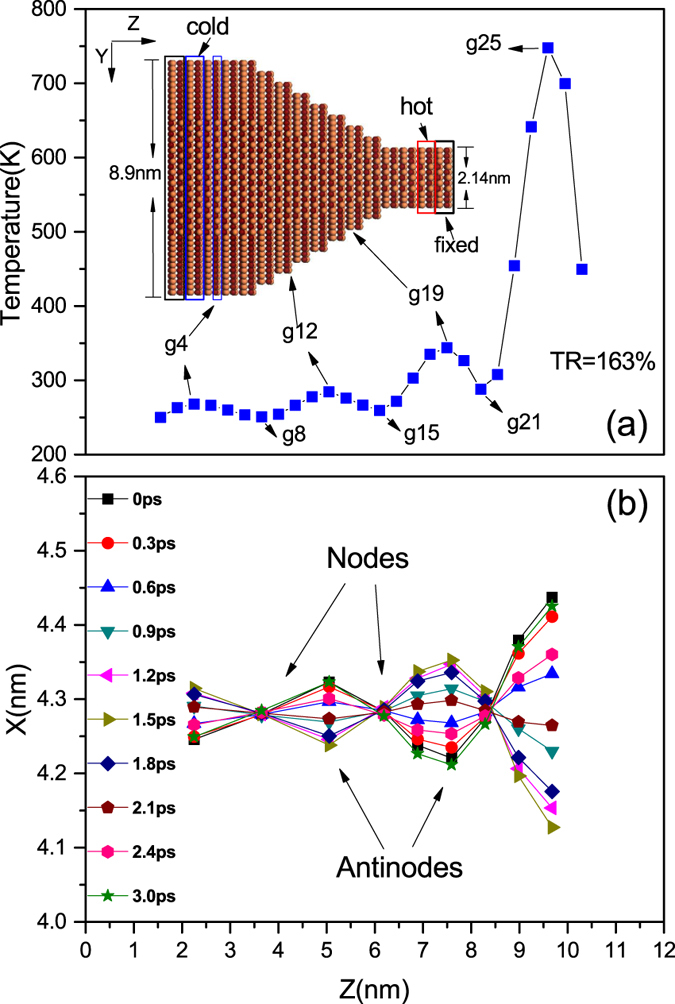
(**a**) Side view of a length-specified (10.93 nm) graded InAs nanowire and the temperature distribution in the wire. The thermal rectification ratio (TR) is also indicated in the figure. (**b**) Trajectory of the coordinate centers of atom groups 4, 8, 12, 15, 17, 19, 21, 23 and 25 during a time period of 3 ps. The alternate distribution of large and small amplitudes suggests the existence of standing wave in the nanowire.

Figure 1(a) shows the temperature profile of a graded InAs nanowire of specified length, in which a typical undulation caused by the existence of the standing waves is clearly observed. Four peaks are seen in this temperature profile, and three of them are connected to the corresponding atom layers (which are also defined as groups) by arrows. To make it self-evident that the periodic undulation is a result of the formation of the standing wave, we obtain the trajectory of the coordinate center of each atom group by tracing the position of each atom. As shown in Fig. 1(b), a typical outline of standing wave can be observed. What’s more, atom groups 8, 15 and 21, which correspond to the temperature valleys shown in Fig. 1(a), move only very slightly over time, indicating that these atoms groups locate at the nodes of the standing wave. In contrast, groups 4, 12, 19 and 25, which correspond to the temperature peaks shown in Fig. 1(a), vibrate periodically with considerable amplitudes, indicating that they are at the antinodes of the standing wave. Such phenomenon is consistent with the equipartition theorem:


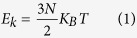


which says that the temperature is directly proportional to the kinetic energy. More importantly, the theorem tells us that the “ultra-high temperature” (higher than the thermal reservoir) is not real temperature but a sign of “high kinetic energy”. Our further investigation on the vibrations of group 19 and group 25 shows that the two atom groups experience a periodic vibration in both the two lateral directions, and their overall orbits are actually quasi-circles.

**Figure 2:**
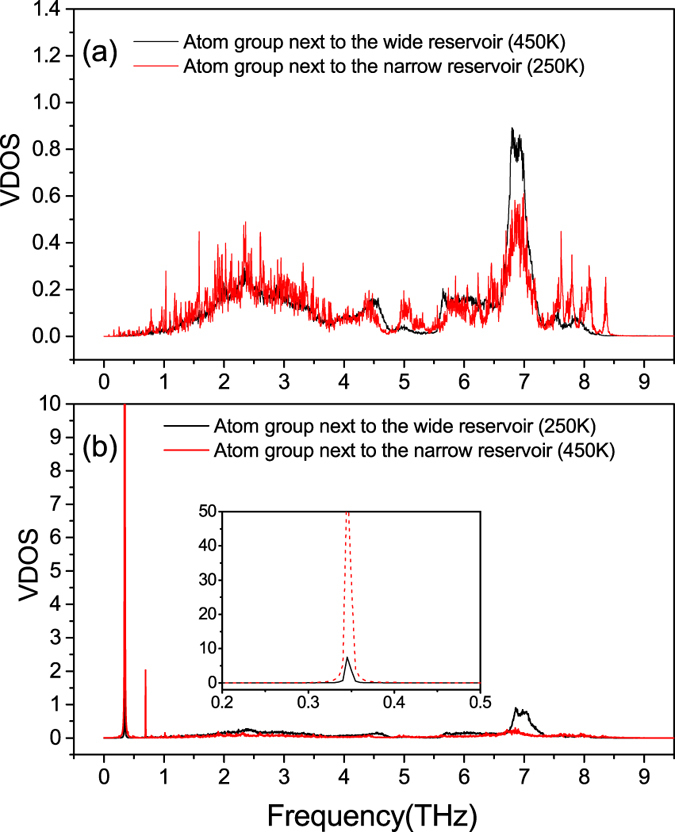
Vibration density of states (VDOS) of the two atom groups next to the thermal reservoirs before and after the temperature difference is inverted.

As for the effect of the standing wave on phonon transport, we find two ways to make it clear. From the point view of real space, the transport of heat are obstructed directly by the significant deformation of the nanowire when standing wave (and resonance, which we will discuss later) appears. The curved nanowire boundary, which can be seen in Fig. 1(b), scatters phonons much more severely than the straight one when the wide end of the nanowire is put into higher temperature and no standing wave exits. However, to have a better understanding of the enhancement of the thermal rectification, we should analyze the issue from the point view of the vibration density of states (VDOS), which can tell us the vibrating frequencies of different part of the structure and has been used to explain thermal rectification effects quite frequently^5^,^25^,^27^,^29^. Figure 2 shows the VDOS of the two atom groups next to the thermal reservoirs before and after the temperature bias is inverted. When the wide end of the nanowire is placed at higher temperature, under which condition no standing wave appears in the nanowire, the VDOS of the atom group next to the hot reservoir and that of the atom group next to the cold reservoir match each other well, and the thermal conduction of the system is thus relatively good. However, when the narrow end is put into higher temperature and standing wave appears, the VDOS of the atom group next to the hot reservoir (narrow) turns into a flat line, except for one very sharp peak and one much weaker peak. In addition, the corresponding frequency of the sharp peak (0.345 THz) is approximately equal to the frequency of the standing wave. This means that the standing wave has dominated the vibration at the narrow end of the nanowire, and higher frequency phonons have been either greatly scattered or suppressed. In contrast, the overall VDOS of the atom group next to the cold reservoir (wide end) is only slightly influenced. In other words, the formation of the standing wave (and resonance) suppresses greatly the number of downstream phonons of different frequencies, and meanwhile enhances the mismatch between the power spectra of the two ends. These two factors, combined with the stationary nature of the standing wave, contribute together to the reduction of the thermal conduction in only one direction and the enhancement of the thermal rectification effect.

**Figure 3:**
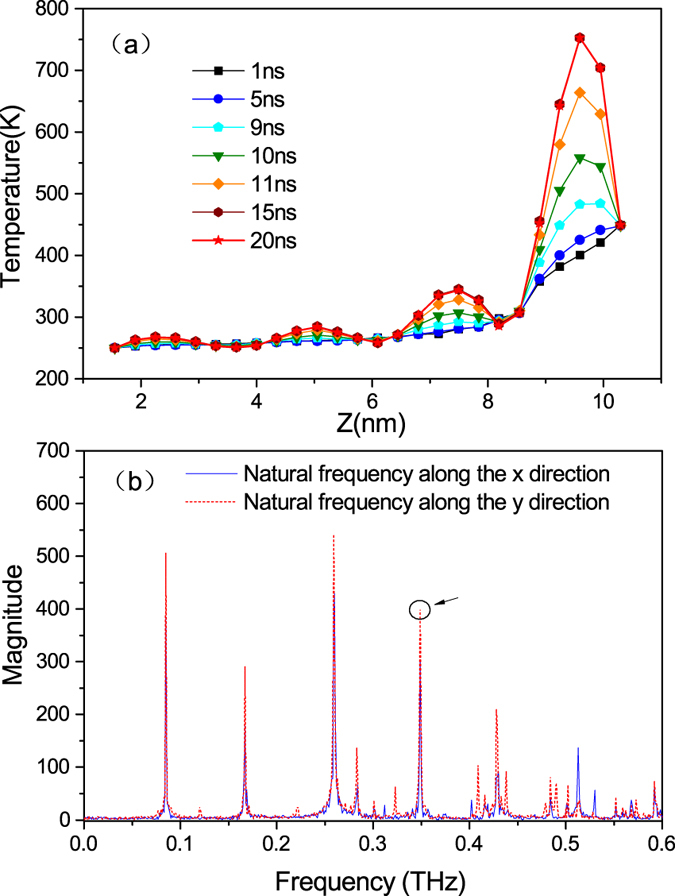
(**a**) Evolution of the temperature distribution in the graded InAs nanowire, which ultimately reaches a steady state. (**b**) Averaged natural frequencies of the graded InAs nanowire along the two lateral directions.

We also manage to reveal the occurrence of resonance in the nanowire by noticing the energy accumulation process in the system. As is seen in Fig. 3(a), the temperature undulation is absent at the first few nanoseconds and the four peaks appear gradually over time. This phenomenon, combined with other incredible facts including the ultra-high temperature (higher than the thermal reservoir) and the large vibration amplitude of the atom groups locate around the antinodes (shown in Fig. 1(b)), provide a picture of resonance in our mind. To test and verify this hypothesis, we calculate the average natural frequencies of one indium atom in group 25 and one arsenic atom that bonding with that indium atom in group 26. The peaks in the frequency spectrum shown in Fig. 3(b) correspond to different orders of natural frequencies of the nanowire. As is seen, the natural frequencies along the X direction and Y direction happen to coincide with each other, and the fourth main peak corresponds with the frequency of 0.349 THz, which is nearly the same with the frequency of the standing wave (0.345 THz). This means that the standing wave is vibrating at one of the natural frequencies of the nanowire, and a resonance process is indeed occurring in conjunction with the formation of the standing wave. Besides, the coincidence of the natural frequencies along the two lateral directions also tells us that strong resonance exists in both the two lateral directions, and that’s why the atom groups that locate at the antinodes of the standing wave are vibrating in quasi-circles.

With the resonance effect being revealed, we can expect to realize a remote energy accumulation process in these asymmetric nanowires. In other words, we can take advantage of the resonance effect around the antinodes of the standing wave to send energy into the inner parts of the nanowire and to make them possess more kinetic energy than their neighbor parts and even the directly heated terminal.

### Standing wave and resonance in asymmetric silicon nanowires

**Figure 4:**
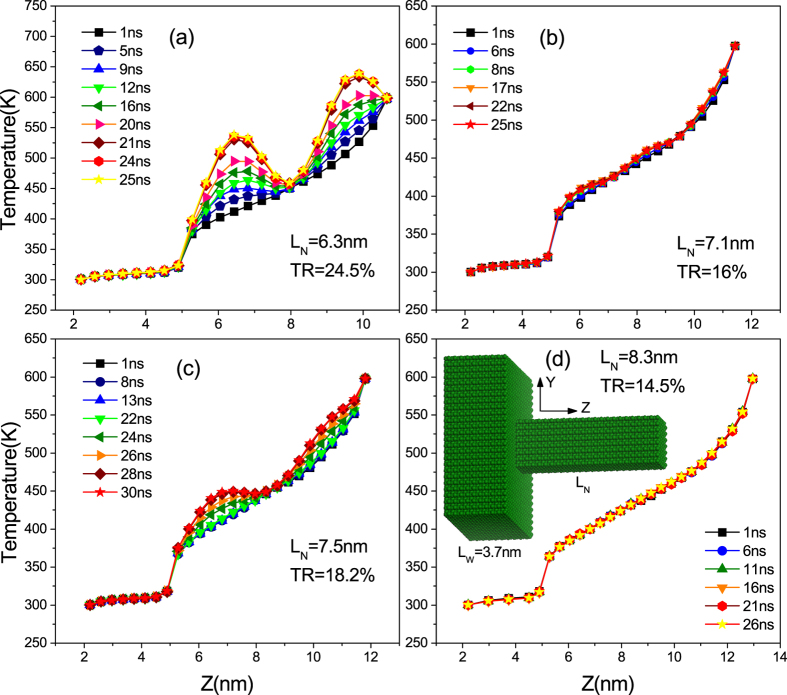
Evolution of the temperature profiles of asymmetric silicon nanowires of different lengths. Sizes and thermal rectification ratios are given in the figure along with a picture of one model, in which the silicon atoms are colored green.

Because we have had a deeper insight into the standing wave phenomenon, we now expect to find it in other materials. To show the generality of the existence of these standing waves in various asymmetric structures, we build a series of two-segment rectangular-bottomed silicon nanowires to fulfill our goal. As shown in Fig. 4, periodic temperature undulations and remote energy accumulation can both be observed in nanowires of various lengths. The sizes and the thermal rectification ratios of the different models are also given in the figure. Specially, for the model in Fig. 4(a), the temperature of the several atom groups near the thermal reservoir is higher than that of the thermal reservoir, just as the case in the graded InAs nanowire we have discussed above, indicating that large amount of kinetic energy has been stored around that antinode. The figure also shows that the temperature behavior of the wide segment of the system is linear, which indicates that no standing wave exists in the wide segment of the structure. We can therefore tell that the wave has been reflected at the interface between the two segments rather than at the fixed left end.

**Figure 5:**
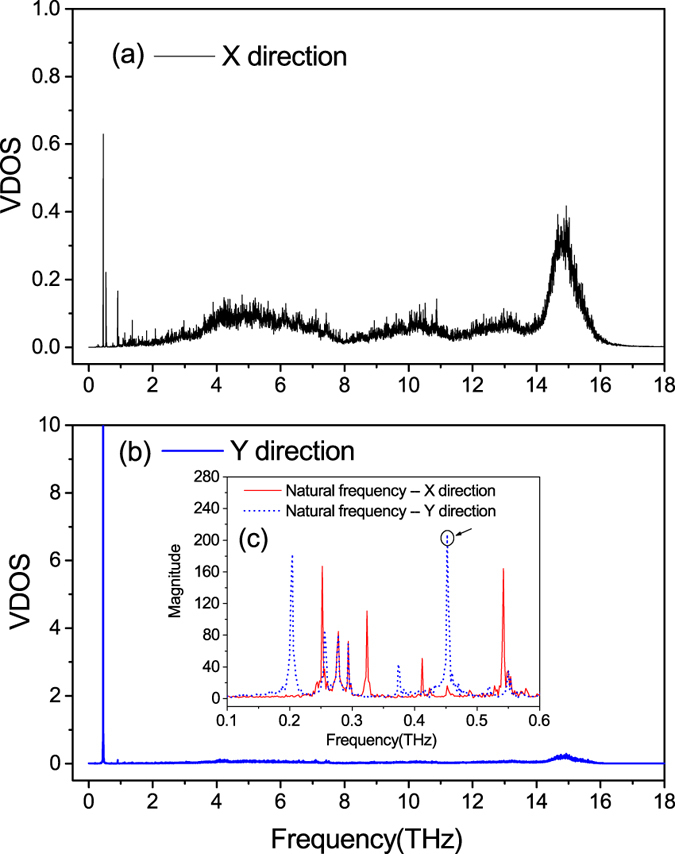
(**a**,**b**) VDOS of the atom group whose temperature is the second peak of the temperature profile in Fig. 4(a). (**c**) Averaged natural frequencies of the atom group.

Inexplicably, however, despite the fact that the thermal rectification effect is better in models where standing waves are formed, and the fact that a stronger resonance leads to better rectification, the rectification ratio that boosted by the combination of standing wave and resonance in these Silicon nanowires (less than 25%) is far less than that in the graded InAs nanowires (163%). To trace the origin of such phenomenon, we check the VDOS of the Silicon nanowire whose temperature profile is shown in Fig. 4(a). To our surprise, only the vibrations along the Y direction have been localized at a specific frequency, as depicted in Fig. 5, while the vibrations along the X direction are only affected very slightly. This means that the distinct standing wave and the strong resonance exist in only the Y direction, which is totally different with the case in the InAs nanowires. Considering that this one-direction standing wave and resonance can not hinder too much energy, we will certainly make sense of the relatively weak effect of the standing wave on the thermal rectification if we can determine the underlying mechanism in this case and subsequently confirm it.

Guided by the conclusion we have drawn above (that the standing wave frequency is one of the natural frequencies of the material), we calculate the average natural frequencies of five atoms (where one atom is in the center and the other four atoms are distributed in the corners) in one specific atom group, whose temperature is the second peak of the temperature profile shown in Fig. 4(a). Surprisingly, as shown in Fig. 5(c), the natural frequencies along the two directions do not always coincide with each other, and at the frequency of 0.452 THz (the standing wave frequency), a very sharp peak and a very small peak are located, representing a major natural frequency along the Y direction and a very minor one along the X direction, respectively. Considering the fact that resonance can stay strong and steady only at the main natural frequency of a structure, the unexpected difference in the natural frequency along the two lateral directions explains well the appearance of the one-direction standing wave and resonance in the asymmetric silicon nanowires. Moreover, the result confirms the validity of our conclusion with regard to the relationship between the standing wave frequency and the natural frequencies of the material.

## Discussion

To analyze the origin of the standing wave and resonance, we must consider the thermal reservoir and the rest part of system separately just as Lee *et al.* did^29^. Although the thermal reservoir is connected with a fixed atom group and thus cannot move freely, it can still vibrate at certain frequencies because most of the atoms in the thermal reservoir are not constrained. What’s more, net momentums result from velocity fluctuations will always exist in subsystems within the thermal reservoir, and the strength of the net momentum is reported to be proportional to (T/N_sub_)^1/2^, where N_sub_ is the number of atoms in the subsystem^29^. Such local vibration makes the thermal reservoir a force provider to the rest part of the structure, and thus turns the whole structure to be a typical forced vibration system. So with the increasing of the temperature in the narrow end, the fluctuating net momentum grows stronger and stronger and begins to shake the rest part of the structure. If the frequency of the net momentum is close to one of the natural frequencies of the nanowire and the distance between the thermal reservoir and the reflecting point is multiples of the half-wavelength, the standing wave and resonance will appear. It is worth pointing out that the net momentum takes effect only when the number of atoms connecting the thermal reservoir and the rest part of the structure (not the number of atoms in the thermal reservoir) is small enough, otherwise the net momentum of different subsystem will average each other out, leaving no forced vibration system. This is why no standing wave and resonance appear when the wide end of the nanowire is put into higher temperature. Last but not least, we prove through calculation that standing wave and resonance will still appear when the thermal reservoir is extended to ten times longer than it is in Fig. 1(a).

By comparing the standing wave and resonance in the two kinds of asymmetric nanowires, we can figure out easily the important role that structural and thermal parameters play in this issue. First, the length of the nanowire determines whether a standing wave can form in the structure; Second, the cross section of the nanowire influences the natural frequencies along the two lateral directions and thus decides whether the standing wave and resonance will appear in one direction only or in both directions; Third, the thermal conductivity (or phonon group velocity) of the material affect significantly the wavelength of the standing wave. For example, the wavelength of the standing wave in the InAs nanowire is longer around the wide end and shorter around the narrow end due to the higher thermal conductivity of the wide bottom, and the wavelength of the standing wave in a carbon nanotube, though not regarded as a standing wave in that work^45^, can be as long as 150 nm. All these factors should be considered in questing for standing wave in asymmetric structures.

In conclusion, we have investigated the standing wave and resonance effect in both graded InAs nanowires and asymmetric Silicon nanowires systematically. The generality of our conclusion is proved, and the uniqueness of each case is revealed. We also analyzed the origin of the standing wave and resonance, and discussed how they manage to obstruct heat transport. Then by pointing out the effects of several structural and thermal parameters on the standing wave, we come to the conclusion that enhanced thermal rectification and strong energy accumulation can be obtained in asymmetric nanowires only when the structure is properly designed.

## Method

Our simulations have been performed on two types of asymmetric nanowire: the first is the wurtzite-phase InAs nanowire with (0001) orientation, which is larger than the InAs-core/GaAs-shell nanowire studied in our previous work, and the second is the zinc-blende-phase silicon nanowire with (110) orientation, which was not studied in our previous work. The diameters of the two ends of the InAs nanowire, which have been designed to be hexagonal, are 8.9 nm and 2.14 nm; and the side-length dimensions of the two ends of the silicon nanowire, which have been designed to be rectangular, are 9.6 nm by 9.1 nm and 2.69 nm by 2.58 nm. It is worth to mention that techniques for growing or fabricating such asymmetric structures, including a monolayer-controlled sculpting technique that has been performed on III-V nanostructures^46^, have been reported and are becoming increasingly mature.

To obtain the temperature profile in each model and the heat current required to calculate the rectification ratios (TR), nonequilibrium molecular dynamics simulations are carried out at the following stages. At the beginning of the simulation, an energy minimization process is performed by iteratively adjusting the atom coordinates. Second, the two outermost atomic layers at each end of the nanowire are set to be freezing to prevent the structure from shifting overall. After that, a Nose-Hoover thermostat^47^,^48^ is used to maintain the temperature at 300 K for 2 ns to equilibrate the system. Then, heat baths are applied at several of the atom layers next to the fixed layers at each end to establish a temperature gradient and a steady heat flow. Finally, time averaging of the heat flux and the temperature is performed for 30 ns to observe the evolution of these quantities and to ensure the accuracy of the exported data.

The VDOS used in this work is calculated from the fast Fourier transform of the velocity autocorrelation function, in which the atom velocities are collected every 25 fs for the 0.25-ns period before the end of the simulation. We also calculated the natural frequencies of the different models using Fourier transforms for the displacements of the atoms over time. The displacements are exported every 50 fs for 1 ns after a 5-ns relaxation of the structure at 300 K. The thermal rectification ratio is defined as:


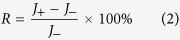


where J_+_ represents the heat current flowing from the wide end to the narrow end, and J_−_ represents the heat current flowing in the opposite direction.

The simulation is mostly carried out using the LAMMPS package^49^ with a time step of 1 fs, and the Tersoff potential^50^, which is well-suited for group III-V and group IV semiconductors, is adopted in the simulation. The parameters required for the InAs nanowires are taken from ref. 51, and those needed for the silicon nanowires are taken from ref. 50. To confirm the reliability of our simulations, we use another group of parameters from ref. 52 to repeat part of the InAs nanowire simulations, and consistent results are obtained.

## Additional Information

**How to cite this article**: Liu, Y.-Y. *et al.* Conjunction of standing wave and resonance in asymmetric nanowires: a mechanism for thermal rectification and remote energy accumulation. *Sci. Rep.* **5**, 17525; doi: 10.1038/srep17525 (2015).
